# Supplementary material for: Insight into the evolutionary assemblage of cranial kinesis from a Cretaceous bird
Source: eLife. 2022 Dec 5;11:e81337. doi: 10.7554/eLife.81337 (PMC9721616; doi:10.7554/eLife.81337)
Supplement: Supplementary file 1. [file elife-81337-supp1.docx]

**Supplementary file 1. Taxa used in geometric morphometric analysis of palatine shape.**

| Taxon | Specimen No. | Source |
| --- | --- | --- |
| *Yuanchuavis* *kompsosoura* | IVPP V27883 | This study |
| *Sapeornis chaoyangensis* | IVPP V19058 | Published photograph (*Hu et al., 2019*) |
| *Archaeopteryx lithographica* |  | Published reconstruction (*Bhullar et al., 2016*) |
| *Ichthyornis dispar* | FHSM VP-18702 | Published CT data (*Torres et al., 2021*) |
| *Hesperornis gracilis* | YPM 1206, KUVP 71012 | Published reconstruction (*Elżanowski, 1991*) |
| *Tragopan caboti* |  | This study |
| *Dromaius novaehollandiae* |  | This study |
| *Alioramus altai* | IGM 100/1844 | Published CT data (*Gold et al., 2013*) |
| *Acrocanthosaurus atokensis* | NCSM 14345 | Published reconstruction (*Eddy and Clarke, 2011*) |
| *Allosaurus fragilis* |  | Published reconstruction (*Madsen, 1976*) |
| *Dromaeosaurus albertensis* | AMNH 5336 | Published reconstruction (*Currie, 1995*) |
| *Erlikosaurus andrewsi* | IGM 100/111 | Published CT data (*Lautenschlager et al., 2014*) |
| *Sinovenator changii* | PMOL-AD00102 | Published CT data (*Yin et al., 2018*) |
| *Incisivosaurus gauthieri* | IVPP V13326 | Published reconstruction (*Xu et al., 2002*) |
| *Velociraptor mongoliensis* | ZPAL MgD-I/97 | Published reconstruction (*Barsbold and Osmólska, 1999*) |
| *Gobivenator mongoliensis* | MPC-D 100/86 | Published reconstruction (*Tsuihiji et al., 2014*) |
